# Strategies for improving the quality of verbal patient and family education: a review of the literature and creation of the EDUCATE model

**DOI:** 10.1080/21642850.2014.900450

**Published:** 2014-04-28

**Authors:** Cara Marcus

**Affiliations:** ^a^ Director of Library Services, Brigham and Women's Faulkner Hospital, Boston, MA, USA

**Keywords:** patient education, communication, interpersonal relations, health literacy, communication barriers, health education, teach-back

## Abstract

*Objective*: Patient and family education includes print, audio-visual methods, demonstration, and verbal instruction. Our objective was to study verbal instruction as a component of patient and family education and make recommendations for best practices for healthcare providers who use this method. *Methods*: We conducted a literature review of articles from 1990 to 2014 about verbal education and collaborated on departmental presentations to determine best practices. A survey was sent to all nursing staff to determine perceptions of verbal education and barriers to learning. *Results*: Through our work, we were able to identify verbal education models, best practices, and needs. We then constructed the EDUCATE model of verbal education, which built upon our findings. *Conclusion*: Verbal education of patients and family members requires a multidisciplinary approach that takes into account learning styles, literacy, and culture to apply clear communication and methods for the assessment of learning. Providers need the skills, time, and training to effectively perform patient and family verbal education every time they care for patients. Further research needs to be performed on how to test, document, and quantify patients' comprehension and retention of verbal instructions.

## 1. Introduction

The need for patient education is widely recognized in the medical community (Behar-Horenstein et al., 2005). Well-educated patients are better able to understand and manage their own health and medical care throughout their lives. Patient–provider communication is a key element of patient education and is often used in conjunction with other teaching practices. Communication is effective when patients receive accurate, timely, complete, and unambiguous messages from providers in ways that enable them to participate responsibly in their care. Patient understanding of information communicated by healthcare providers can lead to enhanced patient satisfaction, better compliance with treatment instructions, improved outcomes, and decreased treatment times and costs (Behar-Horenstein et al., 2005; The Joint Commission, 2010). Patient education is also a requirement for accreditation of healthcare facilities.

In a study of adult patients and visitors enrolled at four Boston-area emergency departments (*N *=* *1010), 24% of participants listed speaking with an expert as their preferred educational modality. That metric was even higher for various demographic groups: 32% of Hispanic respondents and those with less than a high school education preferred verbal education (Kit Delgado, Ginde, Pallin, & Camargo, 2010). Effective verbal patient education has been shown to improve the patient's ability to care for him or herself post-discharge, thus reducing morbidity and mortality. Patient education has also resulted in improvements in the patients' hospitalization experiences, including lessening of pain and anxiety (Montin, Johansson, Kettunen, Katajisto, & Leino-Kilpi, 2010). If improved communication results in better self-care, future medical interventions may be needed less frequently (Kripalani & Weiss, 2006).

Not all patient education is successful. In reality, communication is often partially understood, misunderstood, or misinterpreted. Even with the best of intentions, patient education that fails to educate can lead to adverse events or poor outcomes. The Joint Commission studied patient–provider communication as the root cause of sentinel events and found that poor oral communication caused 10% of these events (The Joint Commission, 2010).

The Brigham and Women's Faulkner Hospital (BWFH) Patient/Family Education Committee set out to explore literature on verbal education and barriers to effective education. Our goal was to share our own internal methodologies and develop a new model of verbal education that included recommendations for best practices for healthcare institutions and providers. While verbal education should be just one part of an integrated, multimodal patient education session, it is vital that it be delivered in a fashion that augments the patient's learning, comprehension, and retention.

## 2. Methods

BWFH is a 150-bed non-profit, community teaching hospital located in Jamaica Plain, Massachusetts. The BWFH Patient/Family Education Committee develops and implements standardized, easily accessible patient education processes and resources in accordance with regulatory requirements. The multidisciplinary committee includes members from nursing, medicine, administration, allied health, nutrition, physical therapy, pharmacy, social work, library services, and the patient population.

In 2010, the committee prioritized initiatives through a brainstorming session, multi-voting process, criteria grid, and impact matrix. Our brainstorming session identified the following projects that our committee saw as priorities at the time:

- Brochure inventory
- Cultural competency
- Educational TV
- Identification of learning styles
- Literacy
- New employee orientation
- Online resources
- Patient education packets
- Patient/Family Resource Center
- Rounding
- Verbal education

Subcommittees were set up within the committee to present the three priority projects selected at the first meeting (inventory, educational TV, and verbal education). The verbal education subcommittee consisted of a physician, a nurse, and a dietitian. The three projects were assessed through an impact matrix for their feasibility, cost not to fix (this includes monetary and public health costs), and impact on the problem.

Verbal education became our initial priority project as the committee discussed that this method was used in every patient–provider encounter and often was used in conjunction with additional forms of education, such as written material. We would address the following focus areas: (1) identifying the learner, (2) assessment of comprehension, (3) continuous education, and (4) documentation of education. We planned to develop guidelines and staff training in these areas.

The committee conducted a literature review of articles from 1990 to present utilizing the following databases: EBSCO CINAHL, GALE InfoTrac Health Reference Center Academic, MD Consult, OVID Journals Database, ProQuest Nursing and Allied Health, and PubMed. Search terms included verbal education, oral education, patient education AND communication, patient education AND oral, patient education AND verbal, physician–patient communication, and nurse–patient communication. We included articles that addressed one or more of the four focus areas identified by the committee. Committee members were asked to volunteer to read one or more articles and report their findings to the group. In addition to the literature review, committee members presented their own approaches to delivery of verbal education within their disciplines (Table 2). The summary of our own approaches was also used to help us formulate a model of best practices. We also conducted an online survey of the nursing staff on patient education practices, which helped determine needs surrounding verbal instruction.

## 3. Review

### 3.1. Effectiveness of verbal education

The literature review identified studies involving verbal education of various demographic groups (children, the elderly, those with hearing impairment, etc.) In addition, studies were conducted surrounding education of patients undergoing treatment for various conditions (cancer, cardiology, orthopedics, etc.).

Posma, van Weert, Jansen, and Bensing (2009) studied 38 patients and found that they wanted to receive concrete information about their disease and treatment, such as diagnosis, prognosis, treatment side-effects, possible complications, and other practical information. This study found that older patients benefited from a question list prepared to discuss subjects during patient education sessions.

Johnson and Sandford (2005) conducted a systematic Cochrane review that compared *written with verbal information* to *verbal information only* in a study of parents of children with health problems. In the two trials selected by the study, findings indicated that parents were more knowledgeable and satisfied with the combination of written and verbal information than verbal education alone. The combination of the information improved parents' scores significantly in terms of knowledge of medications and how to recognize signs of improvement and concern.

In a study of 61 patients (Behar-Horenstein et al., 2005), cardiac patients provided more specific information to their providers about their conditions than general medical patients, resulting in better patient–provider communication. Overall, the patients reported that they received most of their information from verbal interactions they had with doctors and nurses. The majority of the verbal education was perceived by the patients as effective. Nearly three-fourths of the patients (*n *=* *45) stated that they were satisfied with the information they received and the methods hospital staff used to teach them. Only 5% of patients claimed that they received little or no information on signs and symptoms, and only 5% of patients seemed uninformed about their medications.

In a prospective, blinded, randomized, controlled study of 605 patients (Liu et al., 2014), only 9% of patients showed non-compliance with instructions after receiving telephone-based re-education on the day before colonoscopy versus 32.6% of patients who did not receive the verbal re-education.

None of the articles we reviewed developed an approach to quantitatively measure the effectiveness of verbal education; studies have focused on qualitative perceptions of its effectiveness and/or patient satisfaction with education received. Research still needs to be performed on how to test, document, and quantify patients' comprehension and retention of verbal instructions before and after various provider interventions.

### 3.2. Identifying the learner

Patient education is a Joint Commission requirement for hospital accreditation. The hospital provides patient education and training based on each patient's needs and abilities (PC.02.03.01). The hospital performs a learning needs assessment for each patient, which includes the patient's cultural and religious beliefs, emotional barriers, desire and motivation to learn, physical or cognitive limitations, and barriers to communication (EP 1). The hospital respects the patient's right to receive information in a manner he or she understands (RI.01.01.03) (The Joint Commission, 2012).

To offer the highest quality verbal education, a healthcare provider must understand the patient's background, reading level, and how he or she learns best. Different people have different abilities to learn, and providers need to understand what distinct learning preferences and needs the patient may have (Anonymous, 2000; Montin et al., 2010; Posma et al., 2009). After the provider understands the patient's optimal method of learning, he or she can adjust the teaching and training strategy to incorporate many techniques, including demonstrations, diagrams, reinforcement, review, teach-back, support, etc. (Anonymous, 2000).

Cultural, cognitive, and physical differences require different educational approaches (Goody & Drago, 2009). For example, Lieu et al. (2007) recommended that providers communicating with deaf patients should make eye contact, may need to write to communicate, but never assume that there is an exact translation of medical terms into sign language. In another study focusing on hearing-impaired patients, Tye-Murray (1992) empathized that it helps if the provider anticipates hearing loss and the patient may be able to prepare for communication by reading or writing words that he or she wants to know about. For patients whose preferred learning style is not verbal communication, Behar-Horenstein et al. (2005) recommended that providers broaden the use of alternative instructional aids and methods of delivery that utilize auditory, visual, and kinesthetic modalities. Pictures on paper or a screen may serve as simple visual aids to supplement verbal education.

Differences in communication and learning may stem from a variety of factors, including age, gender, ethnicity, or level of education. Elderkin-Thompson and Waitzkin (1999) compared research on communication by men and women as both providers and patients. This study identified a great many differences in communication styles among genders, such as providers communicating more with female patients by giving them more time and using easier terminology. The authors also found that women generally use discussion to clarify explanations, while men often present problems and expect to resolve them. The research also focused on socioeconomic groups; for example, providers usually give more emotional support to poorer patients.

Articles in our review identified roadblocks to education, particularly low literacy (Anonymous, 2000; Owen, 2005). Anyone, even highly literate people, may not be healthcare literate. Providers need to assess patients for their level of literacy prior to providing education. An assessment may include interviews with patient or family members, communication with members of the medical team, or observations of patients (Behar-Horenstein et al., 2005). Providers may be able to assess poor literacy in verbal interactions if the patient asks questions about what has already been explained, asks irrelevant questions, or provides unusual or irrelevant answers to questions (Remshardt, 2011). Another method to identify patients' learning styles is asking what help is needed with understanding medical information (Posma et al., 2009).

### 3.3. Patient–provider communication

When patient education is delivered verbally as part of a multimodal patient education program, the provider assumes the role of the teacher and the patient that of the learner. Richard and Lussier (2007) found that provider–patient discussions have often been regarded as interactive in nature. However, this study found that resulting dialogue mainly consisted of separate monologues, with insufficient real exchange in understanding of the other participant's perspective. The authors described how physicians often adopted the “Information Provider” role in discussions involving medications, and patients the “Listener” role, often significantly limiting their active input. When patients did have some prior knowledge of medications related to their care, discussions with their providers tended to be more interactive, resulting in improved outcomes. Further research is needed to determine what ideal provider and patient communication roles should be. While this may prove elusive, various articles have focused on ways providers can improve communication. For example, Skorpen and Malterud (1997) described communication based on mutual trust.

Talen, Grampp, Tucker, and Schultz (2008) addressed what providers needed from patients to have positive verbal interactions. The patient should have knowledge of his or her medical history and prescriptions and have an attitude that is focused on the treatment plan and the need for follow-up care. Education can be improved by providing supportive staff to help patients and preparing worksheets with questions for the patient to ask in advance. Talen et al. stressed that patients can be *trained* to communicate more effectively with their providers, thus improving their ability to have a conversation that results in better understanding.

### 3.4. Comprehension and retention

Patient education is ineffective if the patient fails to understand what is being taught. However, patients may not even be aware that they do not understand what is being taught to them. In a study of two teaching hospitals (Engel et al., 2009), the majority of patients with comprehension deficits failed to perceive that they had any deficiencies. Only about 20% of patients in this study *reported* comprehension difficulties, but about 78% of the patients *demonstrated* a comprehension deficiency in at least one domain of their visit. Many patients had poor comprehension of multiple aspects of their emergency department care and discharge instructions in this study. Another study by Margolis (2004) found that patients retained about 50% of information by health care providers, and about half of that was remembered correctly.

The ability to comprehend and retain information may decline as patients and family members age; Posma et al. (2009) studied 38 patients and found that older people had more difficulties processing and remembering information than younger ones. Barriers to information retention may also include anxiety, denial, memory deficits, pain, stress, or unfamiliarity (Anonymous, 2000; Margolis, 2004). Talen et al. (2008) found that higher satisfaction with patient–provider communication correlated to the patient's ability to remember his or her provider's recommendations and comply with the instructions. These factors are some of many that may affect the individual's ability to process and comprehend verbal education.

### 3.5. A multidisciplinary approach

Patient and family education should exist throughout the continuum of care. A team of healthcare providers should teach the patient and loved ones about disease management, medications, post-discharge management, and advice on when and how to seek medical attention following hospitalization.

Nurses play a critical role in the education of patients. In a study of more than 400 orthopedic operations, patients who preoperatively visited their nurse reported receipt of more knowledge about their condition than other patients (Montin et al., 2010). A hospital instituted an effective verbal education program where chemotherapy information was provided by oncology nurses during a consultation lasting approximately one hour. A booklet was often used to supplement the verbal consultations. The consultation usually took place two weeks to one day before the first treatment started (Posma et al., 2009). Vreeland, Rea, and Montgomery (2011) conducted an evidence-based review of the literature on heart failure and discharge education. They recommended that the optimal patient education would be a structured, one-on-one session with a specialized registered nurse and repetition of the information by the staff during care.

Physicians provide verbal education during every communication encounter. Richard and Lussier (2007) conducted a descriptive study of medication-related exchanges during 1492 consultations between patients and general practitioners. The authors identified physicians' clinical expertise as technical knowledge that patients do not generally share to any great extent and suggested that physicians can build on patients' own knowledge and experience to increase dialogue.

Many other providers and practitioners play a crucial part in patient education and counseling, including social workers, rehabilitation therapists, home healthcare workers, educators, patient advocates, librarians, etc. Each provider should work individually and collaboratively. Case managers can become facilitators for recognizing gaps in patient education and putting an appropriate plan in place given the patient's needs (Owen, 2005). Behar-Horenstein et al. (2005) surveyed patients about their education and found that about 12% received information from dietitians or pharmacists, with slightly less reporting receiving information from physical therapists or transplant coordinators. Cant and Aroni (2008) stressed that dietitians needed to display a high level of communication competence because the purpose of their communication had the education of a patient as a goal. A hospital developed a scripted patient education tool so that research pharmacists could conduct 45 minute one-on-one patient education sessions (*N *=* *528). Following the patient education sessions, patients were given the opportunity to ask questions or voice concerns. Medication adherence increased to 94.4%, compared to 89.9% in the pre-intervention group (*P* < .0001) (Piazza et al., 2012).

Kruzik (2009) described a team-based initiative that offered a free preoperative program to surgical patients and allowed patients a chance to meet team members, ask questions, and get immediate feedback. This approach led to more positive educational experiences.

The literature also discussed the patient's role in the communication process. Avitzur (2011), medical advisor to the Consumer's Union, identified steps that patients could take to improve communication with their doctor, such as not providing superfluous information and using clear, descriptive language to describe the patient's main problem.

## 4. Models of verbal education

Three verbal education models were identified in our literature review. These models addressed aspects of knowledge, communication competencies, educational methodologies, and communication assessment (Table 1).

**Table 1.** Models of verbal education identified in the literature review.

1. Six dimensions of knowledge	2. Four main communication competencies	3. Suite of tools
Biophysiological	Interpersonal communication	Health information needs
Functional	Nonverbal communication	Health information behaviors
Experiential	Professional values	
Ethical	Counseling skill	
Social		
Financial		

Model 1 (Montin et al., 2010) followed a sample of 123 total joint arthroplasty patients and found that perceived knowledge was highest in the area of biophysiological and functional. Model 2 (Cant & Aroni, 2008) demonstrated that no single skill set operated alone; all were required to be applied in concert to enable effective communication with patients. In their model, they developed specific performance indicators surrounding each competency, such as “Introducing myself to my clients is important” as a component of the professional values competency. The Consumer Health Education Institute (CHEDI) at the University of Virginia developed a model system to create a suite of tools to assist practitioners (1) assess patients' and consumers' personal characteristics and preferences for health information, (2) segment patients and consumers into groups that minimize differences within groups and maximizes differences between groups, and (3) match groups to the health information that most directly meets their needs and preferences (Cohn et al., 2006). CHEDI identified segments of patients, such as those with low literacy, and then developed requirements for health education for those segments, such as avoiding medical jargon.

Table 2 is a summary of presentations given by various departments and services at our institution on their own best practices in verbal education.

**Table 2.** Summary of presentations on success strategies used in practice.

	Home healthcare	Library services	Nutrition
E	Give patients information in small increments, so that the patient can build on each block of information		
D	Teach the patient problem solving skills		
	Try to motivate the patient to gain information, skills, and confidence so that they can make informed decisions about their health		
U		Consumer health library staff can play a role in patient education through the “reference interview” to find out the patient's information needs and learning abilities in order to provide them with resources that they can learn from and share with their providers	
C			Professional tools like “conversation maps” may be helpful in aiding communication
A	Address the patient's current living situation, barriers the patient may be facing in complying with instructions and the patient's motivation and level of confidence		
	Rehabilitation	Social work	Surgery
E	Patients are instructed how to perform exercises and each time they come, the exercise is reviewed and changes are demonstrated		A nurse discusses the surgery with the patient at least one week before. It is also recommended that there should be verbal education early on by the patient's physician. Patients are asked to call in the day before surgery to review the information
			Face-to-face is the best way to communicate because a provider can assess if the patient really understands
D		Try to get an understanding of the patient – how they connect with family, what support systems are in place, and how their environment impacts their care	
		Try to assess if the person is taking in the information presented to them and what stressors are in the way. If they are anxious, try to find out what the source of the anxiety is	
		Try to get the person engaged in conversation and find topics that the patient feels comfortable talking about	
		Try to establish a relationship with the patient	
U			
C	Patients have the opportunity to ask many questions and are given expectations throughout treatment		
A	Visual tools usually supplement verbal education		

Notes: (E) Enhance comprehension and retention; (D) deliver patient-centered education; (U) understand the learner; (C) communicate clearly and effectively; (A) address health literacy and cultural competence.

Based on our literature review and presentations of best practices, the committee developed a process-based model that leads the educator through five stages of verbal education to reach teaching and education goals: the EDUCATE model (Table 3).

**Table 3.** EDUCATE model for verbal education.

E	D	U	C	A	T E
Enhance comprehension and retention	Deliver patient-centered education	Understand the learner	Communicate clearly and effectively	Address health literacy and cultural competence	Teaching and education goals
Use a question list so that patients can ask questions and providers can answer them (Posma et al., 2009)	Talk to – NOT AT – people (Anonymous, 2008; Behar-Horenstein et al., 2005)	Find out what the patient already knows before providing information; ask, “What do you already know about high blood pressure?” (Kripalani & Weiss, 2006)	New communication skills require practice to use them effectively and structured skill development exercises may be helpful for providers. (Kripalani & Weiss, 2006)	Ask patients, “Do you need help understanding health information?” (The Joint Commission, 2010)	Adequate preparation for teaching and learning
Repeat the most important information (Margolis, 2004) and increase the frequency of the message exposure through several repetitions (Ronco, Iona, Fabbro, Bulfone, & Palese, 2012; Takemura et al., 2011)	Practice empathetic skills especially when the view of the patient is different from that of the provider (Cant & Aroni, 2008; Posma et al., 2009; Skorpen & Malterud, 1997)	Be aware of nonverbal messages when delivering verbal communication, including gestures, body language, and dress (Cant & Aroni, 2008)	Present the most important information first (Margolis, 2004). Emphasize one to three key points (Kripalani & Weiss, 2006). Focus on one issue at a time (Avitzur, 2011). Present the information in logical blocks (Remshardt, 2011). Use concrete instructions (Margolis, 2004)	Supplement verbal education with simple written and visual materials (Anonymous, 2008; Behar-Horenstein et al., 2005; Margolis, 2004); however, the materials should be used for reinforcement and not to replace verbal instruction or direct interaction (Anonymous, 2000)	Good teaching methods
Ask patients to repeat information in their own words (Engel et al., 2009)	Ask patients about their life experiences and use to teach (Montin et al., 2010). Use metaphors comparing the patient's care to their life situation (Anonymous, 2008; Behar-Horenstein et al., 2005)	Determine the patient's barriers to health literacy (Paasche-Orlow, 2011). Assessing the ability to learn may include interview or observation (Behar-Horenstein et al., 2005)	Use easy to understand language (Kripalani & Weiss, 2006; Margolis, 2004; Posma et al., 2009)	Use an interpreter if a patient requires one due to language or disability (Dreger, 2001; Lieu et al., 2007). Avoid using technical terminology or medical jargon (The Joint Commission, 2010)	Overcoming barriers to learning
Provide information in several different ways to make sure the patient understands (Skorpen & Malterud, 1997). Audiotapes of patient consultations can be effective for patient recall of verbal education (Friedman, Cosby, Boyko, Hatton-Bauer, & Turnbull, 2011)	Pay attention to the patient's worries and fears and try to dispel them (Posma et al., 2009)	On many occasions family members also need to be educated (e.g. pain management) (Behar-Horenstein et al., 2005)	Patients must be given an opportunity to ask questions prior to discharge. Give them time to speak (Anonymous, 2008; Behar-Horenstein et al., 2005)	A scripted tool may help providers verbalize clearer and more understandable patient education (Piazza et al., 2012)	Teaching as an interactive process
Use the teach-back method (Anonymous, 2008; Kripalani & Weiss, 2006; The Joint Commission, 2010)	Ask patients to state their goals of medical care to begin a discussion (Paasche-Orlow, 2011)	Realize that patients may not even be aware that they do not understand what is being communicated to them (Engel et al., 2009)	Audiotapes of patient consultations can be effective for patient recall of verbal education (Friedman et al., 2011)	Do not just ask the patient, “Do you understand?”. Regardless of their ability to understand, many patients may still answer “Yes” (The Joint Commission, 2010)	Assessment of learning

Note: The last column of the EDUCATE model stands for T and E, Teaching and Education goals, which outlines principles of the model as they relate to the model's individual components.

## 5. Results

In our online survey of nursing staff (*n *=* *46), 81% of the respondents felt that their experience and knowledge were shared verbally as part of the patient and family education process. The survey asked the strengths of patient education at the hospital, the responses (*n *=* *31) included “nurses”, “experienced multidisciplinary staff”, “knowledge base”, “experienced staff”, “the nursing staff”, “nursing staff's expertise”, and “the experience of nurses”.

The survey also identified barriers to verbal education (*n *=* *30) that echoed our findings in the literature review:

- Nurses need more time to listen to and teach patients and families.
- Not all patients are alike.
- Patients' needs are not always known until they are on the unit.
- The timing of education needs to be right.
- When new issues arise, there needs to be a process so the nurse can teach that issue.
- There needs to be communication between surgeons and the family on what to expect after surgery.
- Teaching needs to begin in physician's office at the preoperative visit.
- Caring for a patient alone can be difficult.
- There needs to be a method to follow-up on whether patients really understood what they were taught.

## 6. Discussion

While many articles in our literature review have focused on issues surrounding patient education handouts, classes, video, and online information, we found less research addressing one-on-one verbal education of patients. Verbal education is usually not, and should not, be delivered alone as the only method of patient education. The articles our committee reviewed echoed common themes addressing difficulties encountered in providing and measuring this type of education. While some articles studied specific populations and provided practice recommendations for verbal education to those groups, we found a lack of literature on the ideal verbal education encounter. Measurement of effective verbal education focused mainly on obtaining qualitative patient satisfaction feedback with the instructions received.

The best practices we identified suggest goals providers should strive for when educating patients verbally. Providers should be empathetic and pay attention to patients' fears. Practices like using concrete instructions may be considered common sense, but may be difficult to achieve unless one focuses on doing so. Effective patient education practices need to be learned and reinforced by staff educators in order to become part of the everyday provider care environment. There is a well-known marketing adage called “The Rule of Seven”, which states that someone needs to see or hear your marketing message at least seven times before they take action and buy from you (Hammer & Stanton, 1995).

In their study, Richard and Lussier (2007) concluded that there is no “ideal” patient education conversation. Medical terminology itself is a huge challenge; many of the words that describe medical conditions and treatments are long and multisyllabic and there are no short synonyms. Even seemingly “normal” words, like “diet” may be interpreted differently (e.g. all the calories a person consumes, an organized effort to lose weight, etc.) (Paasche-Orlow, 2011). However, providers can make a dedicated effort to avoid lingo, which may be so ingrained in speech that this will require concentration and willpower.

Two of the more challenging aspects of verbal education are finding out what the patient needs to know (understand the learner) and, after the education has been completed, determining if the patient understood it (enhance comprehension and retention). Providers should conduct health literacy assessments. Certain patients are extremely knowledgeable about their conditions and the consultation can build upon that. An assessment is also a way to find out if there are going to be literacy, disability, or cultural issues. The same education will obviously not apply to every patient. Teach-back tools, such as the Ask-Me-3 program, are recommended for assessing whether the education has been effective. Teach-back includes asking questions to assess what the patient has learned from their education, offering feedback to focus on aspects not understood, and then reevaluating with additional questions to determine if the patient has learned the information (Paasche-Orlow, 2011).

However, even if the patient appears to understand the teaching during a verbal consultation, this does not assure the ability for self-care when the patient goes home. Patients must be able to manage and commit to their own healthcare for the education to be effective (Committee on Patient Safety and Quality Improvement, Committee on Health Care for Underserved Women, American College of Obstetricians and Gynecologists, & Committee Opinion Number 585, 2014). Throughout the patient's medical care, there will be numerous providers teaching the patient, all with different approaches and areas of expertise. Patients need time to understand and absorb the messages their doctors, nurses, and other caregivers provide.

Education also does not necessarily lead to behavioral change. Questions for further research include how one should relate learning to behavioral compliance, and what educators' long-term roles should be in the spectrum of health and wellness.

The ongoing challenge to healthcare organizations is training the frontline staff responsible for educating patients. Programs like the American Medical Association's Health Literacy Train-the-Trainer Program teaches health care professionals how to conduct health literacy programs, which may in turn improve provider education and communication (Maniaci, Heckman, & Dawson, 2008).

## 7. Conclusion

What verbal patient and family education depends upon is the approach and content of patient–provider communication. Our research indicates that there are many complex parameters that influence this communication, such as the patient's learning style, literacy level, culture, environment, etc. By incorporating our simple EDUCATE model into staff education and professional practice, healthcare providers can help guide verbal education to be more patient and family centered. It will be a modification to the providers' current communication styles to incorporate the model's teaching and learning goals into their everyday conversations.

Not all areas of the tool need to be used in all encounters. The EDUCATE model is structured so that providers can apply modules applicable to the individual teaching situation. In its entirety, the model addresses the commonly encountered impediments and obstacles we have identified, as well as provides specific recommendations for modification.

To raise awareness of our findings and educate the hospital staff about our committee's project, we sponsored a presentation on verbal education at a meeting of department heads of our organization and published an article in the hospital newsletter *Faulkner Nurse* (Marcus, 2011). We also piloted a modified Ask-Me-3 handout and poster in our Rehabilitation Department to raise awareness of this type of tool. Ask-Me-3 is a tool developed by the National Patient Safety Foundation that asks three questions: (1) What is my main problem? (2) What do I need to do?, and (3) Why is it important for me to do this? (National Patient Safety Foundation, 2013). After educating patients, providers “teach-back” by asking patients the same questions to test if the learning occurred.

The next steps for our committee are to develop methods of determining whether provider education is comprehended by patients and their families. While our Ask-Me-3 pilot was well received by patients, we realized that we would need a way to document receipt of patient education and change brought about by teach-back tools. We are investigating ways to best document patient education encounters, which will enhance patient care and outcomes beyond the initial encounter and throughout the spectrum of the patient's healthcare continuum.
